# Prevalence, Awareness, Treatment and Control of Hypertension in Nigeria: Data from a Nationwide Survey 2017

**DOI:** 10.5334/gh.848

**Published:** 2020-07-10

**Authors:** Augustine N. Odili, Babangida S. Chori, Benjamin Danladi, Peter C. Nwakile, Innocent C. Okoye, Umar Abdullahi, Maxwell N. Nwegbu, Kefas Zawaya, Ime Essien, Kabiru Sada, John O. Ogedengbe, Akinyemi Aje, Godsent C. Isiguzo

**Affiliations:** 1 Circulatory Health Research Laboratory, College of Health Sciences, University of Abuja, Abuja, NG; 2 Department of Community Health, University of Uyo Teaching Hospital, Uyo, Akwa Ibom, NG; 3 Chukwuemeka Odumegwu Ojukwu University, Awka, Anambra, NG; 4 Department of Medicine, Federal Medical Centre, Gusau, Zamfara, NG; 5 Department of Chemical Pathology, Faculty of Basic Clinical Sciences, University of Abuja, Abuja, NG; 6 Department of Medicine, Federal Teaching Hospital Gombe, Gombe, NG; 7 Department of Medicine, University of Uyo, Akwa Ibom, NG; 8 Department of Human Physiology, Faculty of Basic Medical Sciences, University of Abuja, Abuja, NG; 9 Department of Medicine, University College Hospital, Ibadan, Oyo, NG; 10 Department of Medicine, Federal Teaching Hospital, Abakaliki, Ebonyi, NG

**Keywords:** Prevalence, Awareness, Treatment, Control, Hypertension, Africa, Blood pressure

## Abstract

**Background:**

Previous studies that evaluated the prevalence, awareness and treatment of hypertension in Nigeria were either localized to some specific regions of the country or non-standardized thereby making evaluation of trend in hypertension care difficult.

**Methods:**

We used the World Health Organization (WHO) STEPwise approach to chronic disease risk factor surveillance to evaluate in a nationally representative sample of 4192 adult Nigerians selected from a rural and an urban community in one state in each of the six geo-political zones of the country.

**Results:**

The overall age-standardized prevalence of hypertension was 38.1% and this varied across the geo-political zones as follows: North-Central, 20.9%; North-East, 27.5%; North-West, 26.8%; South-East, 52.8%; South-South, 44.6%; and South-West, 42.1%. Prevalence rate did not differ significantly (p > 0.05) according to place of residence; 39.2% versus 37.5 %; urban vs rural. Prevalence of hypertension increased from 6.8% among subjects less than 30 years to 63.0% among those aged 70 years and above. Awareness was better (62.2% vs. 56.6%; P = 0.0272); treatment rate significantly higher (40.9 % vs. 30.8%; P < 0.0001) and control similar (14 vs. 10.8%) among urban compared to rural residents. Women were more aware of (63.3% vs. 52.8%; P < 0.0001); had similar (P > 0.05) treatment (36.7 vs. 34.3%) and control (33.9% vs. 35.5%) rates of hypertension compared to men.

**Conclusion:**

Our results suggest a large burden of hypertension in Nigeria and a closing up of the rural-urban gap previously reported. This calls for a change in public health policies anchored on a primary health care system to address the emerging disease burden occasioned by hypertension.

## Introduction

In 2016, high systolic blood pressure was reported by the Global Burden of Disease Risk Factors Collaborators as a leading cause of global disease burden in both men and women [1]. In sub-Saharan Africa, emerging epidemiological data suggest that hypertension has become a major public health challenge [2,3].

Wide variation in prevalence, awareness and treatment of hypertension are reported within and between countries of the region [3,4,5,6,7,8]. Various factors ranging from non-standardization of survey methods, use of varying thresholds for diagnosis of hypertension and non-report of age standardized prevalence rates make pooling of the data generated from various studies practically impossible. The overall result is dearth of evidence to inform robust health policies targeted at control of hypertension epidemic in the region.

Nigeria is the most populous country in sub-Saharan Africa and as such her health indices contributes substantially towards defining that of the entire sub-region. The available nationwide data on the epidemiology of hypertension in Nigeria was based on the Non-communicable disease survey published in 1997 [9]. This data is not only obsolete and no longer reliable as the threshold for the diagnosis of hypertension was a blood pressure of 160/95 mmHg as against the acceptable current threshold of 140/90 mmHg. Furthermore, that report did not include data on awareness, treatment and control of hypertension across various regions of Nigeria. Although pockets of regional surveys on hypertension have been conducted in various parts of Nigeria in the past decade, these studies when pooled together to derive national estimates are prone to errors as methodologies and sampling techniques vary widely across them [6,10,11,12,13]. In addition, a critical review of the regional surveys in the light of the national data suggests a distortion in the regional variation earlier documented in the national survey.

Removing the Mask on Hypertension (REMAH) study is a nationwide survey of hypertension aimed at defining the true burden of hypertension in Nigeria. This present report is intended to evaluate the regional and urban-rural variations in the prevalence, awareness, treatment and control of hypertension in Nigeria.

## Methods

### Study Population

REMAH was conducted in 12 communities across six states (one state in each of the six geo-political zones) of Nigeria between March 2017 and February 2018. The general study design and methods have been described in previous details [14]. In brief, we selected participants using a multi-stage sampling technique. The first stage involves random selection of one state from the six geopolitical zones. Guided by the administrative data of the 2015 general elections of the Independent National Electoral Commission (INEC), two (2) local government areas (LGAs) consisting of urban and rural communities were selected in the second stage. We selected state capitals including Abuja Municipal Area Council (North Central), Uyo for Akwa-Ibom (South-South), Ibadan-North for Oyo (South West), Gusau for Zamfara (North-West), Gombe municipal for Gombe (North-East), and Onitsha (the commercial city of the South East) as the urban communities. In the same order, Gwagwalada, Nsit Ubium, Akinyele, Bungudu, Akko and Oyi LGAs were randomly selected for sampling the rural community in these states. In the third and fourth stage respectively, one (1) ward from which one (1) polling unit was randomly selected each from the rural and urban LGAs (Supplementary Table 1). Trained field workers invited consented individuals in each study site to a mobile clinic stationed at a designated location within the community. Using standard methods, they measured blood pressure and anthropometric parameters, and obtained history of previous diagnosis and treatment of hypertension. Out of 4665 adults invited, 4197 consented; giving a response rate of 90%. Five participants had an odd blood pressure reading and were thus excluded from the final analysis.

REMAH study complied strictly with the guidelines for conducting research in human subjects as spelt out in Helsinki Declaration [15]. The University of Abuja Teaching Hospital Health Research Ethics Committee approved REMAH study.

### Diagnosis of Hypertension

Observers received training on blood pressure measurement using the British Hypertension Society Video on Blood Pressure Measurement [16]. They measured brachial blood pressure by auscultation of the Korotkoff sounds at the non-dominant arm, according to the 2013 guidelines of the European Society of Hypertension/European Society of Cardiology (ESH/ESC) [17]. After the participants had rested in the sitting position for at least 5 minutes, the observers obtained five consecutive blood pressure readings at an interval of 30 to 60 seconds. Systolic and phase V diastolic blood pressures were determined to the nearest 2 mmHg. Standard cuffs that had a 12 × 24 cm inflatable portion were used. If the upper arm circumference exceeded 31cm, larger cuffs with 15 × 35 cm bladder were used instead. To address observer dependent errors inherent in the use of mercury sphygmomanometer, we applied three quality control measures previously deployed [14,18,19], to ensure good quality blood pressure measurement. Observers were trained to avoid odd readings, consecutive identical readings and zero end-digit preference. These three parameters were checked periodically and the observers retrained whenever any deviations below the expected standards were observed. A participant’s blood pressure was the average of the five consecutive blood pressure measurements. Hypertension was defined according to the 2013 ESH/ESC guidelines as systolic blood pressure ≥ 140 mmHg and/or diastolic blood pressure ≥ 90 mmHg and/or self-report treatment of hypertension using antihypertensive medications [17]. Among participants diagnosed hypertensive, awareness of hypertension was defined as previous diagnosis of hypertension by a health worker while treatment of hypertension was defined as self-reported use of prescribed medication for management of hypertension. Control of hypertension was defined as systolic blood pressure lower than 140 mmHg and diastolic blood pressure lower than 90 mmHg in a participant receiving treatment for hypertension. The denominator used to determine the control proportion was all hypertensive patients, treated and untreated. Prehypertension was defined according to JNC VII guidelines as systolic blood pressure between 120–139 mmHg and/or a diastolic BP between 80–89 mmHg.

#### Other Measurements

Anthropometric measurements were done using standard methods as described in details previously [14]. Body mass index (BMI) was derived accordingly as weight in kilogram divided by the square of height measured in meters. Field workers administered a modified WHO STEPs questionnaire to collect information on relevant medical history, alcohol and cigarette consumption, and intake of medications.

### Database management and statistical analysis

We used SAS software version 9.4. (SAS Institute, Cary, NC) for database management and statistical analysis. To eliminate bias due to nonresponse and complex survey design (multistaged sampling technique), we standardized the prevalence of prehypertension and hypertension according to the age and sex structure of the 2006 Nigerian population census figures grouped in 10 years category [20]. Adjustment was done by assigning survey weights obtained from this data to each subject (Supplementary Table 1). The overall and subgroup prevalence of prehypertension and hypertension according to age and BMI groups were obtained using PROC SURVEYFREQ. Similarly, PROC SURVEYLOGISTIC was used to test for trend across age and BMI groups. We used mean and SD to report central tendency and spread of data. We compared mean values and proportions of variables of interest between two independent groups using Students t-test and chi-square statistic respectively.

## Results

### Characteristics of participants

Out of 4192 adult Nigerians that had good quality blood pressure readings, 2377 (63.9%) were women, 661 (19.0%) had no formal education and about a quarter (23.8%) were unemployed. The mean age and body mass index (BMI) were 46.7 years and 24.4kg/m^2^ respectively. Overall, about one out of every three (32.2%) consumed alcohol while one out of every thirty participants (3.4%) smoked cigarette. The mean systolic and diastolic blood pressures in the overall population were 129.3 mmHg and 79.8 mmHg respectively as shown in Table 1.

**Table 1:** Socio-Demographic Characteristics of Participants by Geopolitical Zone.

	Overall	N/Central	N/East	N/West	S/East	S/South	S/West

**Number of participants (%)**	4192 (100)	653 (15.6)	1070 (25.5)	709 (16.9)	489 (11.7)	543 (13.0)	728 (17.4)
**Women**	2377 (63.9)	292 (47.5)	600 (57.4)	360 (59.6)	327 (69.1)	303 (61.2)	495 (69.7)
**Work Status**							
**Govt. Employed**	693 (15.2)	110 (22.1)	172 (16.2)	156 (20.3)	66 (10.1)	91 (14.3)	98 (13.9)
**Non-govt. Employed**	315 (5.51)	108 (14.0)	47 (4.35)	17 (1.93)	73 (9.34)	20 (2.08)	50 (7.42)
**Self-Employed**	2110 (55.2)	308 (41.5)	536 (50.2)	214 (35.8)	252 (57.1)	324 (65.6)	476 (63.6)
**Non-Paid**	16 (0.29)	4 (0.54)	2 (0.20)	1 (0.05)	4 (0.33)	2 (0.13)	3 (0.49)
**Unemployed**	1044 (23.8)	122 (21.9)	319 (41.9)	123 (17.4)	92 (23.1)	103 (18.0)	99 (14.6)
**Educational Status**							
**No formal Education**	661 (19.0)	28 (6.90)	250 (24.3)	238 (45.4)	24 (4.72)	19 (3.54)	102 (12.8)
**Primary Education**	903 (25.5)	84 (14.19)	246 (22.6)	85 (13.0)	129 (35.4)	189 (35.2)	170 (22.9)
**Secondary Education**	1157 (25.4)	216 (33.8)	286 (26.4)	171 (20.4)	154 (31.6)	164 (30.5)	166 (23.4)
**Tertiary Education**	1432 (30.1)	316 (45.1)	287 (26.7)	214 (21.2)	175 (23.3)	165 (30.7)	275 (40.8)
**Smoking**	165 (3.40)	50 (5.59)	31 (2.98)	21 (3.20)	18 (5.6)	30 (4.81)	15 (2.03)
**Drinking Alcohol**	1451 (32.2)	348 (47.0)	364 (33.5)	14 (1.79)	230 (56.7)	323 (58.0)	172 (26.0)
**Mean** ± **SE**							
**Age, Years**	46.7 ± 0.1	38.0 ± 0.1	43.8 ± 1.7	40.9 ± 2.3	51.5 ± 2.1	47.0 ± 1.7	50.0 ± 3.2
**SBP, mmHg**	129.3 ± 2.1	118.9 ± 1.5	120.8 ± 5.4	123.9 ± 0.1	136.9 ± 0.3	135.1 ± 0.7	131.5 ± 3.51
**DBP, mmHg**	79.8 ± 0.9	75.5 ± 0.3	75.8 ± 2.0	75.1 ± 0.7	82.3 ± 0.2	83.3 ± 0.4	82.1 ± 0.1
**BMI, kg/m^2^**	24.4 ± 0.6	24.5 ± 0.5	24.3 ± 0.6	22.0 ± 0.1	26.5 ± 1.5	24.2 ± 0.2	24.9 ± 5.7

Systolic and diastolic blood pressures were derived from the average of five consecutive auscultatory readings Govt-Employed = Government Employed; Non-Govt Employed = Non-Government Employed.

### Prevalence of hypertension

Table 2 shows the crude and age-standardized prevalence of hypertension according to sex and place of residence. The prevalence of hypertension overall was 38.1%, 41.8% in women and 31.8% in men. The prevalence rate of 37.5% among the rural dwellers was similar (p > 0.05) to 39.2% among their urban counterparts. Of the six regions, the South-East had the highest prevalence of hypertension (52.8%) while the lowest rate of 20.9% was observed in the North-Central region. Tables 3 and 4 show the prevalence of hypertension according to different categories of age groups and BMI. Prevalence of hypertension increased steadily from 6.8% among participants aged 30 years and below to 63.0% among those 70 years and above (p for trend < 0.0001). Likewise, prevalence also increased from 29.2% among leaner participants with BMI less than 25 kg/m^2^ to 75.1% among those with BMI of 40 kg/m^2^ or more (p for trend < 0.0001).

**Table 2:** Crude and age-standardized prevalence of hypertension according to sex and place of residence.

Region	Sex								Site							

	Men				Women				Rural				Urban			

	N	nHT	Crude %	Adjusted* %	N	nHT	Crude %	Adjusted %	N	nHT	Crude %	Adjusted %	N	nHT	Crude %	Adjusted %

**N/Central**	361	87	24.1	23.0	292	53	18.2	18.6	196	39	19.9	20.0	457	101	22.1	22.1
**N/East**	470	120	25.5	24.3	600	168	28.0	29.9	788	192	24.4	25.0	282	96	34.0	34.2
**N/West**	349	69	19.8	21.5	360	95	26.4	30.4	398	109	27.4	28.2	311	55	17.7	17.5
**S/East**	162	71	43.8	38.0	327	181	55.4	59.5	176	91	51.7	53.1	313	161	51.4	52.2
**S/South**	240	98	40.8	41.1	303	138	45.5	46.8	321	144	44.9	45.1	222	92	41.4	41.6
**S/West**	233	89	38.2	37.5	495	215	43.4	44.1	295	129	43.7	44.4	433	175	40.4	41.1
**Overall**	1815	543	29.4	31.8	2377	850	35.8	41.8	2174	704	32.4	37.5	2018	680	33.7	39.2

N = Number in group; nHT = Number of Hypertensive subjects. * Standardized to the 2006 National Population Census.

**Table 3:** Prevalence of Hypertension according to different age groups.

Region	<30 Yrs		30–39Yrs		40–49Yrs		50–59Yrs		60–69Yrs		>70Yrs	

	No in Group	HT (%)	No in Group	HT (%)	No in Group	HT (%)	No in Group	HT (%)	No in Group	HT (%)	No in Group	HT (%)

**N/Central**	172	5 (4.12)	214	35 (14.9)	155	30 (18.4)	77	16 (24.7)	26	3 (11.0)	9	8 (81.2)
**N/East**	261	7 (2.78)	243	31 (12.9)	208	66 (32.1)	136	57 (42.5)	121	70 (57.8)	101	57 (58.1)
**N/West**	266	18 (6.28)	160	15 (8.24)	116	31 (27.1)	88	44 (45.2)	42	33 (81.5)	37	23 (63.5)
**S/East**	45	6 (12.4)	84	17 (24.1)	119	61 (56.2)	128	87 (67.6)	67	48 (63.1)	46	33 (71.8)
**S/South**	119	16 (12.1)	114	35 (29.2)	116	58 (47.9)	85	53 (65.4)	67	45 (65.4)	42	29 (69.2)
**S/West**	92	6 (6.56)	108	21 (20.8)	147	69 (46.5)	155	83 (54.9)	123	66 (53.6)	103	59 (58.2)
**Rural**	454	21 (6.29)	434	60 (15.9)	410	150 (40.5)	333	155 (51.8)	281	163 (61.0)	262	155 (62.1)
**Urban**	501	37 (7.74)	489	94 (21.7)	451	182 (43.3)	336	201 (61.0)	165	112 (59.9)	76	54 (65.7)
**Overall**	955	58 (6.84)	923	154 (18.1)	861	332 (41.7)	669	356 (55.5)	446	275 (60.6)	338	209 (63.0)

Hypertension was systolic blood pressure ≥140 mmHg and/or diastolic blood pressure ≥90 mmHg and/or use of antihypertensive medication.

**Table 4:** Prevalence of Hypertension according to different BMI categories.

	<25 Kg/m^2^		25-29 Kg/m^2^		30-34 Kg/m^2^		35-39 Kg/m^2^		≥40 Kg/m^2^	

	No in Group	HT (%)	No in Group	HT (%)	No in Group	HT (%)	No in Group	HT (%)	No in Group	HT (%)

**N/Central**	364	43 (10.3)	192	55 (29.7)	73	28 (45.3)	18	12 (70.7)	6	2 (26.7)
**N/East**	665	136 (20.8)	274	86 (32.0)	91	42 (47.2)	31	18 (58.2)	9	6 (68.2)
**N/West**	545	97 (21.5)	126	43 (41.7)	24	12 (47.8)	9	7 (82.2)	5	5 (100)
**S/East**	157	20 (10.6)	166	87 (54.5)	105	65 (65.3)	46	34 (73.9)	15	12 (85.2)
**S/South**	320	109 (36.7)	146	72 (48.6)	56	36 (63.5)	15	13 (95.5)	6	6 (100)
**S/West**	426	154 (36.2)	185	83 (44.1)	80	44 (55.0)	26	16 (61.1)	11	7 (66.3)
**Rural**	1466	384 (30.5)	508	206 (46.8)	143	73 (56.7)	46	34 (79.8)	11	7 (76.2)
**Urban**	1011	209 (28.5)	581	220 (40.3)	286	154 (57.1)	99	66 (62.9)	41	31 (74.6)
**Overall**	2477	593 (29.9)	1089	426 (44.0)	429	227 (56.9)	145	100 (70.2)	52	38 (75.1)

Hypertension was systolic blood pressure ≥140 mmHg and/or diastolic blood pressure ≥90 mmHg and/or use of antihypertensive medication.

### Prevalence of Prehypertension

According to Table 5, about one fifth of the entire study population had prehypertension; 54.5% and 45.5% reside in the urban and rural area respectively. The prevalence of prehypertension was higher among participants aged less than 50 years when compared to the older ones (22.5% vs. 15.4%; p = 0.0334).

**Table 5:** Age Distribution and Prevalence of Prehypertension.

	<30 Yrs		30–39Yrs		40–49Yrs		50–59Yrs		60–69Yrs		>70Yrs		Overall	

	No in Group	PreHT (%)	No in Group	PreHT (%)	No in Group	PreHT (%)	No in Group	PreHT (%)	No in Group	PreHT (%)	No in Group	PreHT (%)	No in Group	PreHT (%)

**N/Central**	172	20 (13.6)	214	32 (17.0)	155	30 (18.4)	77	16 (24.7)	26	3 (11.0)	9	–	653	101 (16.8)
**N/East**	261	44 (17.3)	243	46 (19.4)	208	42 (19.6)	136	34 (24.7)	121	16 (13.2)	101	9 (8.35)	1070	191 (17.8)
**N/West**	266	40 (9.61)	160	33 (18.2)	116	18 (16.4)	88	12 (9.36)	42	3 (5.84)	37	8 (21.9)	709	114 (13.4)
**S/East**	45	6 (9.05)	84	18 (19.1)	119	33 (23.5)	128	12 (8.08)	67	6 (10.7)	46	1 (0.54)	489	76 (12.1)
**S/South**	119	44 (39.2)	114	35 (29.6)	116	32 (27.9)	85	17 (18.0)	67	11 (19.6)	42	6 (13.3)	543	145 (26.2)
**S/West**	92	28 (32.1)	108	36 (32.5)	147	37 (25.6)	155	29 (18.4)	123	24 (20.0)	103	22 (20.3)	728	176 (24.3)
**Rural**	454	72 (16.5)	434	81 (21.0)	410	150 (40.5)	333	62 (14.8)	281	37 (13.3)	262	36 (14.0)	2174	365 (37.5)
**Urban**	501	110 (29.2)	489	119 (29.2)	451	115 (27.6)	336	58 (17.6)	165	26 (21.1)	76	10 (15.5)	2018	438 (24.7)
**Overall**	955	182 (21.3)	923	200 (24.1)	861	192 (23.0)	669	120 (15.9)	446	63 (15.9)	338	46 (14.3)	4192	803 (19.7)

Prehypertension is a systolic blood pressure between 130–139 mmHg and/or diastolic BP between 80–89 mmHg.

### Awareness of Hypertension

**Figure 1:**
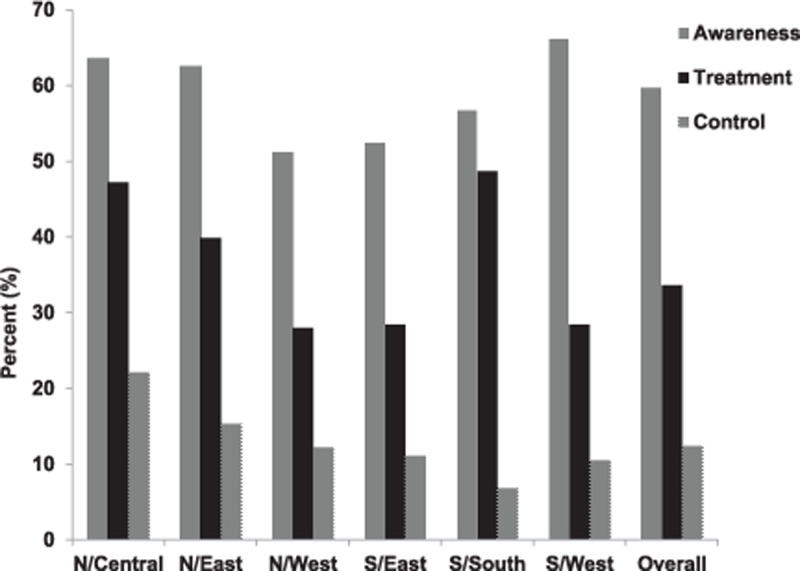
Awareness (vertical lines), treatment (solid) and control (dots) of hypertension across the six geopolitical zones and the overall population. Awareness includes subjects previously diagnosed of hypertension. Treatment includes hypertensives patients on antihypertensive medication and control includes hypertensive patients receiving treatment whose systolic and diastolic BP are less than 140 and 90 mmHg respectively.

Six out of every ten hypertensive patients were aware of their status. More women (65.7%) compared to men (34.3%) were aware of their hypertension status (p = 0002). In comparison to the rural areas, participants in the urban area were more aware (p = 0.0099) of their status (52.2% vs. 47.8%). Figure 1 shows the awareness status according to regions.

### Treatment of Hypertension

About one third of hypertensive patients (33.6%, 95%CI, 33.2–38.4%) were receiving treatment for their condition with no significant difference between women and men (36 vs 34.3%; p = 0.3573). More urban dwellers receive treatment when compared to their rural counterparts (40.9 vs. 30.8%; p < 0.0001). Figure 1 shows the treatment status according to regions.

### Control of Hypertension

Among hypertensive patients, only 12.4% had their blood pressure under control. Overall, control rate among women was similar to men (12.2 vs. 12.5%; p = 0.8696). Likewise, control rate did not differ significantly between the urban (14.0) and their rural (10.8%) counterparts; (p = 0.0727). Figure 1 shows the control rate according to regions.

### Hypertension Care Cascade

**Figure 2:**
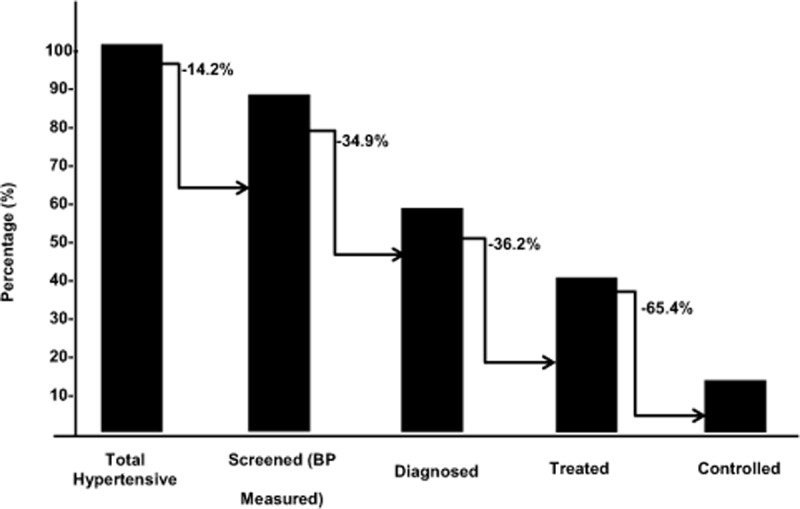
Hypertension Care Continuum Cascade. All hypertensive subjects whether diagnosed or undiagnosed is represented as 100%. Of these, 14.2% have never been screened for hypertension, 34.9% has been screened but not diagnosed, 36.2% have been diagnosed but not receiving treatment and 65.4% were treated but their blood pressure was still uncontrolled.

The care cascade shown in Figure 2 demonstrates significant loss to care at different levels along the hypertension treatment continuum. Of all hypertensive patients, 14.2% had never been screened; while about 35% of those screened remained undiagnosed. Of those diagnosed, 36% were untreated; and among the treated, two-thirds (65.4%) had uncontrolled blood pressure.

## Discussion

The key findings of our study were that 38% of adult Nigerians aged 18 years and above were hypertensive. Out of the hypertensive subjects, 60% were aware of their status, one-third were receiving treatment and 12% had their blood pressure under control. Prevalence of hypertension ranged from 20.9% in the North-Central to 52.8% in the South-East region. Hypertension was as common in the rural as in the urban areas; however, the urban dwellers were more aware of and received treatment for the condition more than their rural counterparts.

Hitherto, the non-communicable disease survey of 1997 (NCD 97) has remained the only available primary nationwide survey data on the epidemiology of hypertension in Nigeria [9]. The survey included 19,997 Nigerians drawn from about 13 states of the country and spread across rural and urban communities. The investigators used a threshold blood pressure of 160/95 mmHg for the diagnosis of hypertension and reported that the prevalence of hypertension was 11.2%, 14% and 9.8% in overall, urban and rural areas respectively. In terms of the geographic spread, the semi-desert area corresponding to the North-West geopolitical region had the highest prevalence of hypertension. Although it is difficult to compare the present study with the NCD 97 because different diagnostic criteria were employed; the narrowed urban-rural gap and the changes in the geographic/regional distribution of the prevalence of hypertension are two aspects that deserve some discussion.

The narrowing urban-rural gap in the prevalence of hypertension may be related to the increasing urbanization of Nigerian rural areas with the attendant shift towards the lifestyle that fuel non-communicable diseases including hypertension [21]. Population growth results in dwindling land for farming thus encouraging rural dwellers to engage in more non-farm economic activities [22,23]. The non-farm activities as opposed to the manual labor driven farm activities encourage sedentary life style and consumption of high-salt processed food. A review of previous studies that evaluated the urban-rural difference in the prevalence of hypertension in South-East Nigeria [24], elsewhere in sub-Saharan Africa [25,26]; Southern America [27], and China suggest either a narrowing of the rural urban disparity or even a trend towards a higher prevalence in the rural compared to the urban areas in some of the studies [11,25,28,29]. Although prevalence of hypertension is obviously increasing in the rural populations of Nigeria, the rate of awareness and treatment has remained significantly higher in the urban as compared to the rural areas. This is consistent across the aforementioned studies conducted in different regions of the world.

Another clear difference between the current report and the 1997 NCD survey is the assertion that the prevalence of hypertension was higher in the semi-desert region compared to the rain forest region or South-East Nigeria. Our report and indeed various other systematic/narrative reviews published in the last decade which tried to analyse data obtained from individual smaller studies carried out in different parts of the Nigeria, agreed that prevalence of hypertension was highest in South-East Nigeria [4,7]. The reason for this epidemiological shift is unknown however; the effect of the Nigerian civil war whose devastating effect was majorly felt in South-East Nigeria may throw some light on this epidemiological puzzle. The war which occurred between 1967 and 1970 witnessed a high level of humanitarian crisis with hunger and famine [30,31], a condition under which most pregnant women had their babies. Children born during this period are now at the middle age of their life, the period during which hypertension and other non-communicable diseases begin to manifest. A growing body of evidence has clearly shown that the root of many NCDs in adults are traceable to unfavourable intrauterine and early childhood period [32,33,34,35]. Hult and Colleagues investigated this line of thought among 1339 adults in a southeastern city of Nigeria [36]. They compared prevalence of hypertension, diabetes mellitus and impaired glucose tolerance among the three groups subdivided based on whether the individual was born before, during or after the war. In comparison with unexposed offspring, being an offspring of a starving pregnant woman, (i.e. those born during the war) was associated with significant increases in the prevalence of hypertension (9.5% vs. 24%) and impaired glucose tolerance or diabetes (8.0% vs. 13%).

Although it is difficult to compare our result with the contemporary data emanating from other African countries due to heterogeneity in study methodology [3,26,37]; our results suggest that the prevalence of hypertension in Nigeria is among the highest in Africa. A review of the 20 countries in Africa who undertook WHO STEPs survey between 2003 and 2009 indicates that the prevalence of hypertension ranged from 8.2 % in Eritrea to 38% in Seychelles [38].

In comparison to the 1997 survey [9], our result suggests that awareness of hypertension increased by about 50% from 33.8% to 60% over a 20 year period. Although this figure is higher than the average of 52.7% reported for the developing countries [39], much lower levels were observed in the rural areas. Despite this seeming progress, this report shows a significant loss along the care continuum highlighting a high rate of unmet needs in hypertension care in Nigeria as only 12% of the hypertensive patients in Nigeria were treated and controlled. This finding is similar to the result of a 2011 health and nutrition survey in South Africa where the authors reported that only 9% of the hypertensive patients were treated and controlled [40]. Several factors ranging from access to health care, poor health seeking behaviour, dysfunctional health system, therapeutic inertia and patients’ non-adherence to prescribed therapies may be responsible for the huge loss in care of hypertension [41,42].

Our results should be interpreted within the context of the potential limitations. Sampling frame from which the participants were drawn was not known as there was no register that contained the entire inhabitants of each community studied. This would have made our sample truly random as the consent would have been given by individuals who had a prior knowledge of their hypertension status. However, we did a thorough community mobilization where the inhabitants were duly educated on the need to participate in the study irrespective of their status. Furthermore, home visits by research assistants encouraged participation. On the whole, a participation rate of 90% was achieved.

A repeat blood pressure measured after at least two weeks apart would have ensured true diagnosis of hypertension according to guideline. We however, averaged five blood pressure readings which approximates more closely to an individual’s usual blood pressure. Furthermore, we deployed standardized methodology to ensure good quality of blood pressure measurement throughout the entire period of the survey.

### Perspectives

This survey came almost two decades after the NCD 97 survey and due to the obvious reasons that have already been discussed [9]; it was difficult to assess the trend in hypertension care, a process that is needed for setting priorities and targets for NCD control. It has been reported that about 28 million of NCD deaths in low and middle income countries are due to weak health systems of which lack of adequate data is a major component. To address this challenge, the “World Health Organization Global Action Plan 2013 to 2020” recommended that member nations should have a stepwise approach to surveillance (STEPS) survey or a comprehensive health examination survey every five years [43]. Nigeria and many other developing countries have not yet attained this target. This can be achieved if the STEPS survey is integrated into the National Health and Demographic survey currently running in Nigeria and other developing countries of Africa. Furthermore, data on hypertension can easily be obtained from volunteers within community groups who would be trained on how to measure blood pressure using semi-automated validated monitors.

## Additional File

The additional file for this article can be found as follows:

10.5334/gh.848.s1 Supplementary Table 1. Multi-staged Sampling of Subjects and Weighting by Design, Age and Sex.
