# Supplementary material for: Prevalence, Awareness, Treatment and Control of Hypertension in Nigeria: Data from a Nationwide Survey 2017
Source: Glob Heart. 2020 Jul 10;15(1):47. doi: 10.5334/gh.848 (PMC7427662; doi:10.5334/gh.848)
Supplement: Supplementary Table 1. — Multi-staged Sampling of Subjects and Weighting by Design, Age and Sex. [file gh-15-1-848-s1.pdf]

Supplementary table 1: Multi-staged Sampling of Subjects and Weighting by Design, Age and Sex

Design Weighting												Age/Sex Weighting				Final Weight	
Strata	Stage 1		Stage 2		Stage 3		Stage 4					Sex				Men	Women
	State	P	LG	P	Ward	P	Polling Unit	P	Base Wt	Age Grp (yrs)	Men*	P	Women*	P			
**S/East**	Anambra	1/5	Oyi	1/17	Awkuzu 4	1/15	Amumobi Village Sq.	1/18	22950	<30	11.8	23/50	13.9	27/50		49,802	42458
		1/5	Onitsha North	1/4	Inland Town 8	1/15	Omaba Pri. Sch	1/17	5100	30-34	4.51	12/25	4. 96	13/25		10608	9792
**S/South**	Akwa-Ibom	1/6	Uyo	1/3	Uyo Urban I	1/11	Fed. Housing Est. (PU:03/31/01/022)	1/22	18480	35-39	3.66	1/2	3.67	1/2		36960	36960
		1/6	Nsit Ubium	1/28	Ubium South II	1/10	Village Sq. Ikot Akamba (PU:03/02/09/007)	1/11	4356	40-44	3.40	53/100	3.06	47/100		8233	9278
**S/West**	Oyo	1/6	Ibadan North	1/9	VN58	1/12	Owo Egbekele	1/36	15552	45-49	2.56	14/25	2.03	11/25		27838	35303
		1/6	Akinyele	1/24	Arilogun/ Eniosa/Aroro	1/12	Igbo Oloyin Village(PU:30/02/03/007)	1/9	23328	50-54	2.36	14/25	1.89	11/25		41757	52955
**N/Central**	FCT	1/7	Municipal	1	Kabusa	1/12	Lugbe FHA Gate(37/03/005/003)	1/14	3500	55-59	1.19	29/50	8.77	21/50		6020	8330
		1/7	Gwagwalada	1/5	Dobi	1/10	Ung. Sarki Dobi/Pri. Sch Dobi (37/03/005/006)	1/10	1176	60-64	1.37	14/25	1.09	11/25		2105	2669

**N/West**	**Zamfara**	**1/7**	**Gusau**	**1**	**Galadima**	**1/13**	**Janyau (PU:36/07/01/017**	**1/45**	**18018**	**65-69 yrs**	**0.63**	**11/20**	**0.52**	**9/20**	**32793**	**40040**
		1/7	Bungudu	1/13	Nahuce	1/11	Dejin Gabas	1/21	3510	⩾70	1.91	71/100	1.48	11/20	4943	6382
**N/East**	Gombe	1/6	Akko	1/2	Garko	1/11	Shongo Hamma (PU:15/01/02/006)	1/52	6480	<30	11.8	23/50	13.9	27/50	14087	12000
		1/6	Kaltungo	1/9	Ture	1/10	Ture Mai (PU:15/07/10/003)	1/12	6864	30-34	4.51	12/25	4.96	13/25	14300	13200

LGA=Local Government Area; P=Probability; Weight=Inverse of Probability; Base Weight=Product of Weights Across Four Stages of Design; Final Weight=Base Weight × Sex Weight. Final weight shown for each site is weight for only one age group; *=Number in millions
